# Comparative Analysis of Antimicrobial Resistance in Male Uropathogens Before and After the COVID-19 Pandemic: A Single-Center Study from Romania

**DOI:** 10.3390/medicina62050889

**Published:** 2026-05-05

**Authors:** Răzvan-Ionuț Popescu, Răzvan-Cosmin Petca, Cristian Mareș, Aida Petca, Michael Bassil, Viorel Jinga

**Affiliations:** 1 Faculty of Medicine, “Carol Davila” University of Medicine and Pharmacy, 8 Eroii Sanitari Blvd., 050474 Bucharest, Romania; razvan-ionut.popescu@drd.umfcd.ro (R.-I.P.); cristian.mares@drd.umfcd.ro (C.M.); michaelbassil31@gmail.com (M.B.);; 2 Department of Urology, “Prof. Dr. Th. Burghele” Clinical Hospital, 20 Panduri Str., 050659 Bucharest, Romania; 3 Department of Urology, “Saint John” Clinical Emergency Hospital, 13 Vitan-Barzesti Str., 042122 Bucharest, Romania; 4 Department of Obstetrics and Gynecology, CF2 Clinical Hospital, 63 Marasti Blvd., 011464 Bucharest, Romania

**Keywords:** urinary tract infection, male patients, antimicrobial resistance, Gram-negative, Gram-positive

## Abstract

*Introduction*: Urinary tract infections (UTIs) in male patients are a topic that has received less attention in the medical literature. Current management strategies recommended by most guidelines are largely based on research involving female populations, which limits their applicability to men, in whom UTIs are often considered complicated. While the COVID-19 pandemic has brought about many changes in antibiotic treatment, this study aims to compare antimicrobial resistance patterns of uropathogens in male patients between the COVID-19 pandemic and post-pandemic periods. *Materials and Methods*: A retrospective descriptive study including urine-culture positive cases in male patients was conducted at a tertiary-level university urology center in Bucharest, Romania. To assess temporal trends, the analysis used four selected six-month intervals during the COVID-19 pandemic (2020–2022) and the post-pandemic period (2023–2025). Inclusion was limited to adult male patients aged at least 18 years who had a single identified pathogen and significant bacteriuria (at least 10^5^ CFU/mL). Duplicate and polymicrobial samples were excluded. In accordance with CLSI guidelines, bacteria were identified and antimicrobial susceptibility was assessed using standard microbiological methods. Statistical analysis was made using Python 3.11.3. *Results*: A total of 3158 urine positive urine cultures from male patients were included. *Gram-negative* isolates were the most frequent, with *E. coli* being the most common urinary pathogen, followed by *Klebsiella*. The most common *Gram-positive* isolate was *Enterococcus*. Antimicrobial resistance in *Gram-negative* pathogens were higher in the post-pandemic period compared to the pandemic period, particularly to amoxicillin-clavulanic acid, and levofloxacin, with carbapenem resistance exceeding 20%. *E. coli* showed increased resistance rates to levofloxacin, and amoxicillin-clavulanic acid, and ceftazidime. Resistance of *Klebsiella* spp. exceeded 30% for imipenem and meropenem. Resistance to amoxicillin-clavulanic, ceftazidime, and imipenem acid increased in *Proteus* spp. Even though *Pseudomonas *spp.demonstrated higher resistance rates to several antibiotics, no statistical differences were observed. *Enterococcus* spp. showed a stable profile, demonstrating resistance to levofloxacin, penicillin, and ampicillin. *Conclusion*: Among male patients, uropathogens’ antimicrobial resistance was higher in the post-pandemic period compared to the COVID-19 period, particularly among Gram-negative bacteria. Regarding empirical therapy, there are significant concerns regarding the rise in resistance to antibiotics such as fluoroquinolones and β-lactams, as well as the emergence of resistance to carbapenems.

## 1. Introduction

Urinary tract infections (UTIs) are among the most frequently diagnosed bacterial infections in both hospitalized and outpatient settings and are among the most pressing concerns of the modern era. The latest epidemiological studies reveal over 150 million new cases worldwide each year, making this a major healthcare issue with significant socio-economic impact [1,2]. The prevalence risk of developing UTIs increases with advancing age and increasing comorbidities [1,2]. Despite numerous advisories to limit antibiotic treatment, unnecessary prescriptions remain common in clinical practice [3]. Although some progress is already visible, antimicrobial resistance remains a major concern [4]. While urinary tract infections exhibit considerable variability in prevalence and AMR, they place a heavy burden on healthcare systems.

Most of the actual guidelines’ recommendations for antibiotic administration are based on the incidence, prevalence, and resistance rates observed in female patients. Besides the fact that UTIs in males are less frequent and mostly associated with advancing age, this should not be underestimated as long as they are mostly associated with several risk factors, including functional and anatomical anomalies of the urinary tract, prostate involvement or recent instrumentation. The European Association of Urology (EAU) states that antibiotic administration should be based only on diagnostic accuracy in male cohorts [5].

Several sex differences were identified and implicated in the natural evolution of urinary tract infections. While lifetime prevalence in females is higher, according to well-known risk factors such as a shortened urethra and perineal proximity, urinary tract infections in males are less frequent but are mostly classified as complicated due to underlying pathologies [1,6].

Regarding bacterial prevalence, *E. coli* represents the leading uropathogen in both sexes. While recent data reveal alarming rates of bacterial resistance, this phenomenon appears to be more pronounced in elderly male patients, especially those who require urethral catheterization or urinary tract instrumentation [2]. The major difference between sexes is that females are most likely to develop cystitis and pyelonephritis, while males often present with prostatic involvement [7].

Antibiotic course administration also differs between males and females, suggesting that males often require longer courses and that recurrence rates differ. Some hormonal and behavioural factors were associated with higher recurrence rates in uncomplicated cystitis, whereas the same scenario in male patients should be closely investigated to assess early structural lesions or chronic bacterial prostatitis [5].

The COVID-19 pandemic, which began in 2019, has led to important conclusions regarding the epidemiology and evolution of UTIs, especially among hospitalized male patients. The frequently reported secondary bacterial infections, especially in severely ill patients, were associated with prolonged hospitalization and the need for invasive devices [8,9]. Reported studies showed higher rates of catheter-associated UTIs during 2020–2022 than in pre-pandemic surveillance data [10]. Sex and age were found to be key factors responsible for worse SARS-CoV-2 prognostic outcomes and highlighted the risk of nosocomial infections in male patients [11].

One of the most important concerns during the recent pandemic, especially in the beginning, was the increased use of antibiotics, which has led to higher rates of antimicrobial resistance among hospital uropathogens [12]. For more than 25 years, the Infectious Disease Society of America (IDSA), has been continuously warning about the importance of determining local resistance profiles, as long as urine cultures are not consistently recommended by primary care providers, which would be the best option for optimizing antibiotic administration [13].

Romania was recently highlighted by the European Centre for Disease Prevention as one of the highest-antibiotic-resistance countries, and, more worryingly, as one of the countries with increased antibiotic consumption. This should serve as an important warning to the medical community regarding the responsibility of judicious antibiotic administration [14].

Although recent government policies on recording and monitoring antibiotic use have aligned Romania with World Health Organization (WHO) recommendations, there are still problems in monitoring bacterial prevalence and resistance rates. Considering that most of the guideline antibiotic treatment recommendations are made based on female-reported resistances, in males, there is still a gap in data [15]. Given these concerns, the main objective of this study was to compare the antimicrobial resistance patterns among male uropathogens between the COVID-19 pandemic and post-pandemic periods. The secondary objective was to assess the differences regarding bacterial prevalence between the mentioned periods.

## 2. Materials and Methods

### 2.1. Study Design and Population

The present study is a descriptive retrospective analysis including male patients from whom urine samples were collected at a highly representative tertiary urology center and academic institution in Bucharest, Romania: the “Prof. Dr. Th. Burghele” Clinical Hospital (BCH). Given that the first part of 2020 was significantly affected by the COVID-19 pandemic, including periods of lockdown and reduced hospital activity, September 2020 was selected as the starting point to ensure a stable, representative patient population. To achieve consistent comparisons between COVID-19 pandemic and post-pandemic while minimizing the impact of seasonal variability, the analysis was structured into four predefined six-month intervals (September–February), each encompassing autumn and winter seasons. This approach has been used in previous epidemiological and antimicrobial resistance surveillance studies assessing defined pandemic and post-pandemic periods [16,17,18]. The intervals were selected to allow comparison between two periods during the COVID-19 pandemic (2020–2022) and the post-pandemic period. The included intervals were: 1 September 2020–29 February 2021, 1 September 2021–28 February 2022, 1 September 2023–29 February 2024, 1 September 2024–28 February 2025. The study included only positive urine cultures from male patients during the selected intervals. All urine samples processed in the hospital microbiology laboratory during the specified periods were reviewed. Duplicate isolates from the same patient were excluded, based on the personal identification number. Only the first positive urine culture was registered.

**Figure 1:**
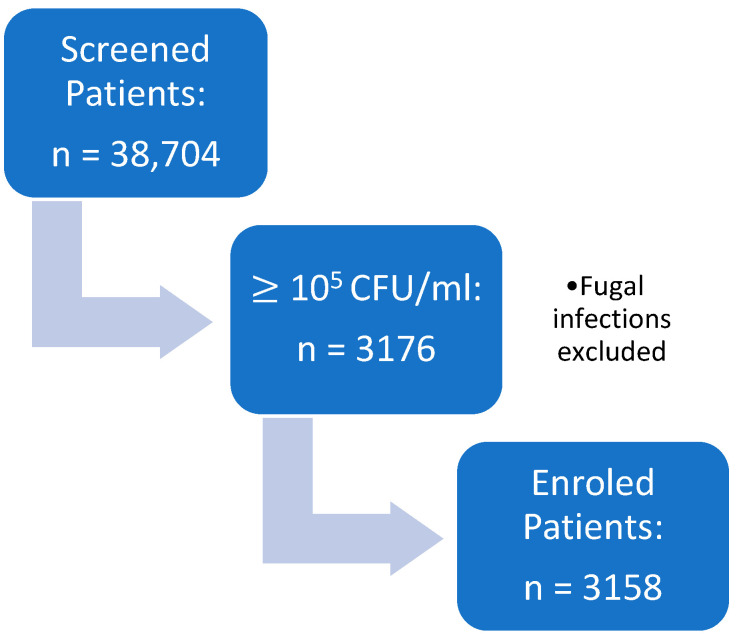
Graphical representation of patients assessed and included in the study group.

Despite the constraints imposed by the COVID-19 pandemic, the hospital activity continued without any restrictions after the lockdown. Owing to its extensive infrastructure, with over 135 urology beds, the institution served as a major referral center for non-COVID urological care, ensuring the uninterrupted management of patients with urological infections throughout the pandemic. A graphical representation of the patients’ selection process can be observed in Figure 1. 

In all cases, the patients included in the study were either hospitalized or received outpatient care. Consequently, it was not possible to collect a more detailed medical history about associated pathologies, of administered medical treatment, and its evolution for the non-hospitalized group. Furthermore, it is not possible to distinguish between recurrences and infections diagnosed for the first time.

The study was conducted in accordance with the ethical principles outlined in the Declaration of Helsinki for research involving human subjects. All procedures involving patient data were performed in accordance with institutional and national ethical standards, and patient confidentiality was strictly maintained.

### 2.2. Inclusion and Exclusion Criteria

Inclusion criteria:

1. Positive Urine Test ≥ 10^5^ CFU/mL;
2. Single bacteria identified on culture;
3. Age ≥ 18 years;
4. Male patients.

Exclusion criteria:

1. Urine test < 10^5^ CFU/mL;
2. Female Sex;
3. Multiple bacteria presence on culture;
4. Duplicate urine culture results from the same patient during the analyzed period (based on the personal identification number, only the first positive urine culture was registered);
5. Patients with urinary catheters.

The antibiotic treatment policy was established in accordance with the annually revised European Association of Urology guidelines [15]. According to its specifications, the minimum duration of the antibiotic regimen was preferred based on a careful evaluation of the specific disease. If the treatment was started empirically, it was properly adjusted based on urine test results. For hospitalized patients, the hospital also benefits from the Infectious Disease department’s advice whenever needed.

Regarding antibiotic susceptibility testing, all urine samples were incubated for 24 h after inoculation onto standard plates. Columbia sheep and lactose agar were the most frequently used for bacterial growth. In specific instances, the Chapman medium was used for *Staphylococcus* spp. Bacterial growth exceeding 10^5^ CFU/mL in no more than 2 isolates was considered significant. The antibiotic inhibition zone evaluated by the Kirby-Bauer disk diffusion technique was analyzed according to the Clinical and Laboratory Standards Institute (CLSI) guideline for Antimicrobial Susceptibility Testing (AST) [19]. All bacterial identification and susceptibility testing were previously reported in several studies conducted in our department [19,20,21].

### 2.3. Statistical Analysis

Data were collected and structured using Microsoft Excel (version 2016, Microsoft Corporation, Redmond, WA, USA). Descriptive statistical analyses were performed in Python version 3.8.9, using JupyterLab 3.2.4 and the Pandas library 1.3.4. Categorical variables were expressed as absolute frequencies and percentages. Data comparison between groups regarding antimicrobial resistance rates was assessed using the chi-square test; when expected cell counts were fewer than 5, Fisher’s test was applied. A *p*-value < 0.05 was considered significant for the compared cases. Grammarly and DeepL were used for language accuracy.

## 3. Results

After analyzing the demographic parameters of the studied population, patients were divided into 2 age groups based on the 55-year landmark, which many authors consider the starting point for the manifestation of lower tract urinary symptoms related to increasing UTI prevalence. Focusing on the age distribution, the present results showed a higher prevalence of urinary tract infections among elderly, vulnerable patients. Table 1 shows that most cases occurred in patients over 55 years old in both groups. The mean age distribution showed similar patterns in both groups (70.5 years in the pandemic group vs. 69.4 years in the post-pandemic group).

Regarding the distribution of bacterial pathogens across the two analysed periods, *Gram-negative* bacteria showed the highest prevalence, with similar results in both groups, as illustrated in Table 2. *E. coli* continues to be the most prevalent diagnosed uropathogen among male patients, closely followed by *Klebsiella* spp. Higher rates of evolution were observed in *Pseudomonas* spp. during the post-pandemic period (10.90%) compared with the pandemic period (7.26%), suggesting a potential shift toward a higher risk of healthcare-associated UTIs. *Enterococcus* spp. remained the most frequently isolated pathogen among *Gram-positive*.

This study focused on determining the antimicrobial resistance rates in both *Gram-negative* and *Gram-positive* isolates. Table 3 and Table 4 illustrate the overall resistance rates registered for both bacterial groups.

The overall resistance rates among *Gram-negative* pathogens showed alarming increases for amoxicillin-clavulanic acid. (43.24% vs. 50.37%), levofloxacin (39.36% vs. 44.55%) compared with the pandemic period. Another important finding was the increasing resistance to last-resort antibiotic classes, such as meropenem and imipenem, which both showed over 20% resistance in the post-pandemic period. Similar trends, but lower values, were also observed when analysing ceftazidime (21.83% vs. 27.11%) and aztreonam (22.8% vs. 31.54%), suggesting worsening susceptibility to β-lactams. One of the most stable regarding antimicrobial resistance seems to be fosfomycin, and amikacin is still effective against all *Gram-negative* bacteria in both predefined periods.

Among *Gram-positive* bacteria, resistance patterns were stable, with some selective variations in both pandemic and post-pandemic time frames. Levofloxacin has shown the same concerning resistance in both the pandemic and post-pandemic periods. Despite showing alarming resistance to many antibiotic classes, *Gram-positive* bacteria remain sensitive to vancomycin, confirming its therapeutic role as a viable option. Overall, the *Gram-positive* bacteria revealed minimal differences between the analyzed periods in contrast to the *Gram-negative* bacteria.

*E. coli*, the most frequently identified uropathogen, was higher in the post-pandemic period compared to the pandemic period to one of the most commonly used antibiotics, such as levofloxacin, amoxicillin-clavulanic acid, and ceftazidime (Table 5). The AMR for levofloxacin rose from 37.24% to 45.57%, while, similarly, for amoxicillin-clavulanic ac. it rose from 33.81% to 40.13%. Even though the ceftazidime maintained resistance rates under 20%, a significant difference and an increasing trend were observed (12.04% vs. 17.21%).

Preserved sensitivity was observed for carbapenems, amikacin, and fosfomycin.

**Table 5:** *E. coli* resistance and sensitivity rates.

Antibiotic	*E. coli* R		*E. coli* S		*E. coli* R		*E. coli* S		*p* Value
	Pandemic				Post-Pandemic				
	No.	%.	No.	%.	No.	%.	No.	%.	
Amikacin	23	4.69	467	95.31	24	4.82	474	95.18	1.00
Amoxicillin- Clavulanic Acid	164	33.81	321	66.19	191	40.13	285	59.87	0.049
Aztreonam	60	12.99	402	87.01	25	19.23	105	80.77	0.098
Trimethoprim/ Sulfamethoxazole	3	23.08	10	76.92	193	45.09	235	54.91	0.196
Ceftazidime	59	12.04	431	87.96	85	17.21	409	82.79	0.027
Fosfomycin	7	1.55	444	98.45	5	1.07	461	98.93	0.728
Imipenem	4	0.83	479	99.17	2	0.83	238	99.17	1.0
Levofloxacin	181	37.24	305	62.76	221	45.57	264	54.43	0.010
Meropenem	3	0.63	474	99.37	0	0.0	51	100.0	1.0
Nitrofurantoin	48	13.33	312	86.67	39	9.58	368	90.42	0.128

The second most frequent isolated bacteria, *Klebsiella* spp., showed a notable increase in AMR in the post-pandemic period to nitrofurantoin, rising up to 68.51% (Table 6).

The most important increased resistance rate after COVID-19 was observed in imipenem and meropenem, exceeding 30%, highlighting a potential emergence of resistance to last-resort carbapenems.

**Table 6:** *Klebsiella* spp. resistance and sensitivity rates.

Antibiotic	*Klebsiella* R		*Klebsiella* S		*Klebsiella* R		*Klebsiella* S		*p* Value
	Pandemic				Post-Pandemic				
	No.	%.	No.	%.	No.	%.	No.	%.	
Amikacin	64	16.71	319	83.29	86	22.11	303	77.89	0.07
Amoxicillin- Clavulanic Acid	222	57.81	162	42.19	223	63.35	129	36.65	0.144
Aztreonam	135	38.35	217	61.65	45	39.13	70	60.87	0.969
Trimethoprim/ Sulfamethoxazole	8	36.36	14	63.64	144	44.17	182	55.83	0.622
Ceftazidime	139	36.2	245	63.8	145	37.47	242	62.53	0.771
Imipenem	56	15.09	315	84.91	60	32.09	127	67.91	<0.001
Levofloxacin	159	41.3	226	58.7	160	42.67	215	57.33	0.757
Meropenem	55	14.4	327	85.6	26	33.33	52	66.67	<0.001
Nitrofurantoin	110	56.7	84	43.3	124	68.51	57	31.49	0.02

When analyzing *Pseudomonas* spp., although major increases in resistance rates over the COVID and post-COVID compared periods were observed for antibiotics such as levofloxacin (48.11% vs. 49.39%) and amikacin (31.13% vs. 28.98%), no significant differences were observed (Table 7).

Last-resort antibiotics showed a relatively stable trend over time, but their resistance patterns remain a major concern, with over 30% resistance.

**Table 7:** *Pseudomonas* spp. resistance and sensitivity rates.

Antibiotic	*Pseudomonas* R		*Pseudomonas* S		*Pseudomonas* R		*Pseudomonas* S		*p* Value
	Pandemic				Post-Pandemic				
	No.	%.	No.	%.	No.	%.	No.	%.	
Amikacin	33	31.13	73	68.87	51	28.98	125	71.02	0.803
Aztreonam	37	36.27	65	63.73	52	43.33	68	56.67	0.351
Imipenem	36	33.64	71	66.36	52	37.14	88	62.86	0.663
Levofloxacin	51	48.11	55	51.89	81	49.39	83	50.61	0.935
Meropenem	37	33.64	73	66.36	51	32.28	107	67.72	0.919

In contrast to *Pseudomonas* spp., in *Proteus *spp.a significant increase in resistance was observed for amoxicillin-clavulanic ac., rising from 34.27% during the COVID-19 pandemic to 53.21% in the post-pandemic period (Table 8). Similarly, resistance to ceftazidime increased significantly from 10.81% to 22.31%, while imipenem was a particular concern, showing a significant increase from 5.56% to 20.0% in a small number of cases.

**Table 8:** *Proteus* spp. resistance and sensitivity rates.

Antibiotic	*Proteus* R		*Proteus* S		*Proteus* R		*Proteus* S		*p* Value
	Pandemic				Post-Pandemic				
	No.	%.	No.	%.	No.	%.	No.	%.	
Amikacin	6	4.03	143	95.97	9	7.14	117	92.86	0.468
Amoxicillin- Clavulanic Acid	49	34.27	94	65.73	58	53.21	51	46.79	0.002
Aztreonam	9	6.38	132	93.62	1	4.0	24	96.0	0.599
Trimethoprim/ Sulfamethoxazole	5	71.43	2	28.57	64	60.95	41	39.05	0.599
Ceftazidime	16	10.81	132	89.19	27	22.31	94	77.69	0.012
Imipenem	8	5.56	136	94.44	7	20.0	28	80.0	0.049
Levofloxacin	51	34.93	95	65.07	49	39.84	74	60.16	0.408
Meropenem	4	2.78	140	97.22	3	6.52	43	93.48	0.344

After assessing the antibiotic susceptibility profile of *Gram-negative* uropathogens, the antibiotic susceptibility profile of the primary *Gram-positive* isolate was also examined. *Enterococcus* spp., the most frequent pathogen in its class, revealed relatively stable results when comparing the pandemic and post-pandemic situation (Table 9). Although no significant differences were observed, several antibiotics exhibited an alarming resistance profile. The most representative resistance profile was observed in levofloxacin (48.7% vs. 47.89%), followed by penicillin (32.26% vs. 38.64%), and ampicillin (14.5% vs. 13.06%).

A significant, albeit small, increase was observed for vancomycin (0.35% vs. 2.87%).

**Table 9:** *Enterococcus* spp. resistance and sensitivity rates.

Antibiotic	*Enterococcus* R		*Enterococcus* S		*Enterococcus* R		*Enterococcus* S		*p* Value
	Pandemic				Post-Pandemic				
	No.	%.	No.	%.	No.	%.	No.	%.	
Ampicillin	39	14.5	230	85.5	35	13.06	233	86.94	0.720
Fosfomycin	10	3.57	270	96.43	14	5.53	239	94.47	0.377
Levofloxacin	131	48.7	138	51.3	136	47.89	148	52.11	0.915
Linezolid	5	1.82	270	98.18	11	4.1	257	95.9	0.186
Nitrofurantoin	12	4.26	270	95.74	17	6.27	254	93.73	0.382
Penicillin	90	32.26	189	67.74	102	38.64	162	61.36	0.143
Vancomycin	1	0.35	285	99.65	8	2.87	271	97.13	0.040

Regarding *Staphylococcus* spp., resistance rates remained stable over the analyzed periods, with no statistically significant differences between periods. Although stable, there are nevertheless concerning data regarding resistance to levofloxacin (58.33% vs. 54.05%) and penicillin (Table 10).

**Table 10:** *Staphylococcus* spp. resistance and sensitivity rates.

Antibiotic	*Staphylococcus* R		*Staphylococcus *S		*Staphylococcus* R		*Staphylococcus* S		*p* Value
	Pandemic				Post-Pandemic				
	No.	%.	No.	%.	No.	%.	No.	%.	
Trimethoprim/ Sulfamethoxazole	24	40.0	36	60.0	24	34.29	46	65.71	0.623
Levofloxacin	35	58.33	25	41.67	40	54.05	34	45.95	0.748
Linezolid	1	1.56	63	98.44	2	2.82	69	97.18	1.0
Nitrofurantoin	1	1.82	54	98.18	3	4.48	64	95.52	0.756
Penicillin	47	78.33	13	21.67	62	84.93	11	15.07	0.448

## 4. Discussion

### 4.1. General Data Comparison

The characteristics of the study population can partially explain the distribution of pathogens in this study. Since the patient analysis was conducted at a reference urology centre in Romania and included only male patients, a higher rate of healthcare-associated infections is expected. Within these institutions, the bacterial distribution may differ from that found in the community, where the recorded percentage of *E. coli* exceeds 60%. Typically, studies conducted in in-hospital patients demonstrate a higher incidence of opportunistic bacteria, such as *Klebsiella*, *Enterococcus*, and *Pseudomonas* [2,5,20].

Recent epidemiological data on a higher prevalence of UTIs in older patients confirm the mean age of 70 years old reported in the present study. Numerous recent studies have demonstrated an association between age and risk factors such as benign prostatic hyperplasia, urinary retention, urethrovaginal catheterization, institutionalization, and comorbidities, with a significant increase in the incidence of urinary tract infections [1,2,21,22]. According to recent analyses based on male subjects, patients over the age of 60 are at high risk for developing UTIs, especially those who are hospitalized or require urological care [15,21,22,23]. Taken together, these results confirm, the advanced age of the patients included in this study is consistent with current demographic trends regarding the prevalence of UTIs.

### 4.2. Overall Bacterial Comparison to Previous Results

The high prevalence of *Gram-negative* bacteria observed in this study confirms recent epidemiological data demonstrating their high prevalence in both community-acquired and nosocomial infections. Among the *Enterobacterales*, *E. coli* in particular is the most common pathogen implicated in the development of UTIs [2,20].

Although nitrofurantoin and fosfomycin demonstrate very good susceptibility rates, similar to other recent studies showing their excellent therapeutic activity in the treatment of urinary tract infections, in male patients, they are more difficult to use, as they are more suitable for the treatment of uncomplicated infections [24,25]. Furthermore, antibiotics such as carbapenems and amikacin maintain good susceptibility rates against *Enterobacterales*, as demonstrated by numerous global surveillance studies.

Recent studies, in accordance to our findings, reveal an alarming worldwide trend of bacteria in developing resistance to the most frequently used antibiotics, especially for fluoroquinolones and β-lactams [20,25].

Data reported over the past 10 years from patient have indicated a gradual increase in resistance to fluoroquinolones among *E. coli* and other *Enterobacterales* bacteria. This increase has most often been attributed to the overuse of these antibiotics, due to their efficacy [2,23].

Studies conducted during the post-pandemic setting have indicated increases in antibiotic resistance rates to the common bacterial agents, but also in last-line resort drugs like carbapenems, particularly among *Gram-negative* bacteria [26]. The data obtained in this study are consistent with research conducted during and after the COVID-19 pandemic and confirm that inappropriate antibiotic use in hospitalized patients and the increased rate of prolonged hospital stays are correlated with rising resistance rates, particularly for urinary tract infections [8,10].

This study also aligns with recent studies regarding resistance rates for *Gram-positive* bacteria, particularly *Enterococcus*. The antimicrobial resistance rates are high for the most commonly used antibiotics; sensitivity to antibiotics such as vancomycin, linezolid, nitrofurantoin, or fosfomycin remains [27]. Even though fosfomycin and nitrofurantoin retain activity against *Gram-positive* bacteria, they have limited utility in men, as they are primarily used to treat uncomplicated infections in women [24,27].

The overall data obtained in this study for both *Gram-negative* and *Gram-positive* bacteria are consistent with the general findings reported in recent specialized studies, which indicate alarming rates of resistance to fluoroquinolones and aminopenicillins. Furthermore, the relatively good susceptibility rates for fosfomycin, nitrofurantoin, and carbapenems are also consistent with recent studies on the activity of antibiotics against various categories of urinary pathogens [2,20,24,25].

Findings from current data and specialized literature underscore the importance of optimizing antibiotic treatment strategies based on local prevalence data and of continuously monitoring resistance trends across various population groups [2,23,28].

### 4.3. Gram-Negative Comparison

*Escherichia coli*, the most commonly isolated pathogen, shows a marked increase in antibiotic resistance worldwide, particularly in healthcare-associated infections and among patients with high antibiotic use [29,30]. Both in the present study and in international epidemiological studies, *E. coli* exhibits an alarming resistance rate to some of the most commonly used classes of antibiotics, such as fluoroquinolones and β-lactams. Another concerning aspect regarding resistance to β-lactam antibiotics is that global epidemiological data indicate an increasingly widespread prevalence of bacteria producing extended-spectrum β-lactamases (ESBLs) [30].

Regarding susceptibility data in both the present study and other recent studies, *E. coli* demonstrated favourable susceptibility profiles for aminoglycosides, fosfomycin, and carbapenems. This indicates that these classes of antibiotics remain effective despite the rising trend in resistance rates.

For *Klebsiella* spp., this study demonstrated increased resistance rates after the COVID-19 pandemic not only to fluoroquinolones but also to antibiotics such as nitrofurantoin and carbapenems. These alarming data registered in the post-pandemic period are also consistent with the current trend in the resistance spectrum of *Klebsiella* reported in previous studies [31]. Increased resistance to last-line antibiotics such as carbapenems in *Enterobacterales* caused by carbapenemase-producing bacteria is becoming an increasingly serious problem worldwide, due to the severe limitations in treatment options [32].

Another major focus of this study was the increasing rates of antibiotic resistance among *Pseudomonas* spp. across most antibiotic classes registered in both COVID and post-COVID period. Although no major changes were observed between the two periods analysed, the resistance rates are consistent with data from recent studies that identify this bacterium’s ability to develop resistance through intrinsic mechanisms such as efflux pumps, reduced membrane permeability, and biofilm formation [33]. All these mechanisms and processes significantly reduce Pseudomonas’ susceptibility to aminoglycosides, fluoroquinolones, cephalosporins, and carbapenems, making this bacterium a significant threat regarding urinary tract infections [33].

Compared with the pandemic period *Proteus* spp. antimicrobial resistance rates to the most common classes of antibiotics was higher, while maintaining relatively good susceptibility to aminoglycosides and carbapenems. These data correlates with current epidemiological data demonstrating that this bacterium retains good sensitivity to these classes [34].

All patterns of bacterial resistance and sensitivity over the compared periods, of *Gram-negative* organisms analyzed in this study correlate with international epidemiological data, demonstrating that these uropathogens, which account for approximately 90% of all urinary tract infections, exhibit increasing rates of AMR worldwide. This also highlight the necessity for rapid improvement in local studies for choosing the proper empiric antibiotic treatment [28,29,30,31,32,35].

Gram-negative uropathogens showed higher antimicrobial resistance in the post-pandemic period compared to COVID period findings, particularly among Enterobacterales.

Resistance to last-resort agents, including carbapenems, was higher in several *Gram-negative* isolates, raising concern for carbapenemase-producing organisms. These patterns may reflect some aspects during pandemic period regarding increased antibiotic usage, prolonged hospitalization, and higher rates of healthcare-associated infections. Overall, the findings suggest that *Gram-negative* resistance trends in the male population may have been influenced by COVID-19.

### 4.4. Gram-Positive Comparison

*Enterococcus* spp., the most commonly identified *Gram-positive* uropathogen, was shown in this study to exhibit low antibiotic resistance rates in both compared periods to nitrofurantoin, fosfomycin, and linezolid—antibiotics cited in the literature as viable therapeutic options for the treatment of urinary tract infections [27,36]. Unfortunately, some of these have limited efficacy in the male population due to poor penetration into the prostate [15]. As in other studies that identified minimal resistance rates, ampicillin retains its antibacterial activity against *Enterococcus* spp. [36,37]. Another aspect that should not be overlooked is the slight increase in vancomycin resistance, which aligns with the global trend of increasing vancomycin-resistant enterococci (VRE), particularly in settings where antibiotic administration and prolonged hospitalization contribute to this [37,38].

Resistance data levofloxacin, which showed extremely high but stable rates in this study in both COVID and post-COVID settings, are consistent with the general susceptibility data published in recent years for these pathogens [27,37].

In the present study, *Staphylococcus* spp. demonstrated adequate susceptibility to antibiotics such as vancomycin and linezolid. These findings are consistent with existing data in the specialized literature, as it is well known that these antibiotics are used as effective therapies for the treatment of *Gram-positive* bacteria, including methicillin-resistant *Staphylococci* [39,40]. The increase in resistance to ampicillin identified across multiple specialized studies may indicate widespread production of β-lactamases among these bacteria [40,41]. The data obtained in this study, in line with recent epidemiological data, indicate that although fluoroquinolones and older β-lactam antibiotics exhibit high resistance rates, *Gram-positive* bacteria nevertheless retain good susceptibility to classes such as vancomycin and linezolid [36,37,38,39,40,41].

Gram-positive uropathogen isolates showed relatively stable antimicrobial resistance patterns across the pandemic and post-pandemic periods, suggesting a more limited impact of COVID-19–related factors compared to Gram-negative bacteria. A slight increase in vancomycin resistance among enterococci may reflect the selective pressure associated with increased antibiotic use and prolonged hospitalizations during the COVID-19 period. Overall, these findings indicate that, while the pandemic influenced antimicrobial use, its effect on Gram-positive resistance patterns remained relatively modest.

### 4.5. Further Directions

Most international organizations, such as the World Health Organization, and the European Antimicrobial Resistance Surveillance Network, are continuously sounding the alarm regarding the importance of following guidelines for antibiotic prescribing and monitoring their use, as well as implementing training campaigns for prescribing physicians to reduce rates of uropathogen resistance [5,42]. The resistance patterns of *Gram-negative* and *Gram-positive* bacteria identified by this study underscore the importance of conducting prevalence and resistance studies that are both local and gender-specific, as these may reveal significant differences.

Another immediate need would be to improve national surveillance systems through the adoption of appropriate government policies. The European Antimicrobial Resistance Surveillance Network seeks to provide epidemiological data to identify resistance patterns and to adjust antibiotic treatment administration based on clear evidence regarding the prevalence of uropathogens [2,42].

Furthermore, government measures should have a greater impact on healthcare sectors. In urological practice, measures to prevent infections should be taken by improving catheter management and implementing standardized protocols [5,10].

Perhaps one of the most pressing issues regarding bacterial resistance is investment in research into new classes of drugs and possibly rapid diagnostic techniques, which will be essential in the very near future [2,10].

### 4.6. Strengths and Limitations

This study has numerous strengths, and one of the most important is that it includes a cohort of over 3000 urine culture-positive cases analyzed in a male population over a 4-year period. This facilitates a thorough assessment of bacterial resistance patterns.

Another important point to note is that this study included comparative data from the COVID-19 period with data from the post-pandemic period. Throughout this entire period, the hospital operated at full capacity after lockdown period during the pandemic situation as a treatment center for non-COVID patients, performing a wide range of urological surgical procedures without restrictions, covering both oncological conditions and lithiasis or benign pathologies. Given its high patient volume as a referral centre, outpatient cases are also very numerous, with the hospital serving patients from over one-third of the country’s territory.

The hospital also has an Infectious Diseases Department responsible for managing newly diagnosed cases of UTI. To our knowledge, this is the largest study to date conducted on a male population in Romania.

The present study also has some limitations, the most important of which are its retrospective nature and the inclusion in the database of both hospitalized and outpatient patients. In addition, given the inclusion of non-hospitalized cases, the study cannot provide a breakdown of the prevalence of various pathologies within the study cohort. Given that only the first positive urine culture was registered, this study can’t provide data about the recurrence of UTIs. A further limitation is the use of six-month intervals instead of a complete comparison over a whole year, which may lead to seasonal bias. Also, the hospital urology profile as a tertiary center may lead to higher registered resistance rates than in the general population due to the fact that complex pathologies are treated. The exclusion of urinary catheters is also another important limitation of the present study. Last but not least, the study’s single-center design means that the results cannot be accurately generalized to the country’s population.

## 5. Conclusions

In this single-center study of male patients, antimicrobial resistance patterns differed between the COVID-19 pandemic and post-pandemic periods, with higher resistance rates observed in the post-pandemic group, particularly among *Gram-negative* uropathogens. *Enterobacterales*, especially *Escherichia coli* and *Klebsiella* spp., demonstrated higher resistance rates to commonly used antibiotics such as fluoroquinolones and β-lactams, while preserved activity was noted for fosfomycin, aminoglycosides, carbapenems. Notably, increased resistance to carbapenems in the post-COVID setting raises concern regarding the emergence of highly resistant bacterial strains. In contrast, Gram-positive organisms showed relatively stable resistance profiles between the two compared periods. To the best of our knowledge, this is one of the largest studies evaluating the antimicrobial resistance evolution after the COVID-19 situation in the Romanian male population These findings highlight the importance of continuous local surveillance and support the use of culture-guided therapy when managing urinary tract infections in male patients.

## Figures and Tables

**Table 1:** Age distribution.

	<55 Years No./%.	>55 Years No./%.	Total
Group 1 (Pandemic)	173/11.21	1370/88.78	1543
Group 2 (Post-pandemic)	174/10.77	1441/89.22	1615

**Table 2:** Bacterial prevalence in the analysed groups.

		Pandemic No.	Pandemic %.	Post-Pandemic No.	Post-Pandemic %.
*Gram -*	*E. coli*	507	32.86	520	32.20
	*Klebsiella*	400	25.92	397	24.58
	*Pseudomonas*	112	7.26	176	10.90
	*Proteus*	154	9.98	129	7.99
*Gram +*	*Enterococcus*	300	19.44	299	18.51
	*Staphylococcus*	67	4.34	77	4.77
Other		3	0.19	15	1.06

**Table 3:** *Gram negative* (*Gram -*) Overall resistance rates.

Antibiotic	*Gram—*R		*Gram—*R		*Gram—*R	
	Pandemic		Post-Pandemic		Overall	
	No.	%.	No.	%.	No.	%.
Amikacin	126	11.17	170	14.3	296	12.78
Amoxicillin- Clavulanic Acid	441	43.24	473	50.37	914	46.66
Aztreonam	241	22.8	123	31.54	364	25.16
Trimethoprim/ Sulfamethoxazole	16	38.1	402	46.69	418	46.29
Ceftazidime	246	21.83	318	27.11	564	24.52
Fosfomycin	13	2.73	14	2.8	27	2.76
Imipenem	104	9.41	121	20.1	225	13.18
Levofloxacin	442	39.36	511	44.55	953	41.98
Meropenem	99	8.89	80	24.02	179	12.38
Nitrofurantoin	171	29.58	167	27.97	338	28.77

**Table 4:** *Gram negative* (*Gram +*) Overall resistance rates.

Antibiotic	*Gram +* R		*Gram +* R		*Gram +* R	
	Pandemic		Post-Pandemic		Overall	
	No.	%.	No.	%.	No.	%.
Amikacin	4	6.9		5.56	5	6.58
Ampicillin	39	14.39	35	13.06	74	13.73
Trimethoprim/ Sulfamethoxazole	24	39.34	25	29.76	49	33.79
Fosfomycin	12	4.2	14	5.49	26	4.81
Levofloxacin	166	50.46	176	49.16	342	49.78
Linezolid	6	1.77	13	3.83	19	2.8
Nitrofurantoin	13	3.86	20	5.92	33	4.89
Penicillin	137	40.41	164	48.66	301	44.53
Vancomycin	1	0.34	8	2.87	9	1.57

## Data Availability

Data supporting the reported results are available from the authors.
